# Internet Dietary Guidance for Pediatric Inflammatory Bowel Disease: A Quality and Readability Crisis

**DOI:** 10.7759/cureus.102398

**Published:** 2026-01-27

**Authors:** Rachel A Donaldson, Nicole F Miller, Bruno P Chumpitazi, John L Lyles

**Affiliations:** 1 Pediatric Gastroenterology, Duke University School of Medicine, Durham, USA

**Keywords:** crohn's disease, diet, internet, nutrition, pediatric inflammatory bowel disease, readability, ulcerative colitis

## Abstract

Background: Diet and nutritional therapy are treatment options for children with inflammatory bowel disease (IBD). Parents of children with medical conditions often turn to the internet for medical guidance. However, the quality and readability of internet dietary information for pediatric IBD are currently unknown. The objective of this study was to evaluate the quality and readability of websites about diet for pediatric IBD.

Methods: Top internet websites for the searches “IBD, diet, children,” “Crohn’s disease, diet, children,” and “ulcerative colitis, diet, children” were rated using the DISCERN instrument, a validated tool for rating consumer health information, on a scale of 1-5 to assess reliability, information quality, and overall quality (5 = highest reliability or quality). The Flesch-Kincaid grade level (FKGL) was used to determine website readability.

Results: The mean reliability scores were 3.1 for searches on “IBD, diet, children,” 3.0 for “Crohn’s disease, diet, children,” and 3.1 for “ulcerative colitis, diet, children.” The corresponding mean information quality scores were 2.5, 2.5, and 2.4, and the mean overall quality scores were 2.8, 2.7, and 2.9. The mean reading grade levels required to understand the content per FKGL were 11.9, 10.8, and 11.1. Across the websites, 23 highly variable dietary recommendations were made.

Conclusions: For internet search results about diet and pediatric IBD, mean scores indicated moderate website reliability but poor information quality and overall quality. Websites were written at approximately an 11th-grade reading level, above the recommended standard for patient education. Dietary recommendations were numerous and inconsistent.

## Introduction

Inflammatory bowel disease (IBD), including Crohn’s disease and ulcerative colitis, is a chronic condition involving inflammation of the gastrointestinal tract. IBD is often diagnosed in childhood or adolescence, with around 25% of patients diagnosed before 20 years of age [1]. Pediatric IBD affects between 100 and 200 per 100,000 children in the United States, and incidence has been increasing over time [1,2]. Pharmacologic treatment options for maintenance include 5-aminosalicylic acid (5-ASA), immunomodulators, biologics, and small molecules [3]. However, environmental factors, including diet, may play a role in the pathophysiology of IBD [4].

Dietary treatments may benefit patients with IBD [5,6]. Recommended dietary regimens for IBD have included the Mediterranean diet, Specific Carbohydrate Diet (SCD), Crohn’s Disease Exclusion Diet (CDED), exclusive enteral nutrition diet (EEN), and others [7,8]. Each diet has a different emphasis, and certain diets are recommended during different states of IBD (e.g., stricture and inflammation) [7]. The Mediterranean diet is a plant-focused diet emphasizing a variety of whole grains, fruits, and vegetables [7]. The SCD restricts hard-to-digest carbohydrates while emphasizing a nutritionally complete grain-free diet, low in sugar and lactose [8]. In comparison, the CDED is a whole foods diet designed to limit foods that may adversely alter the intestinal barrier and affect the microbiome (e.g., emulsifiers and highly processed foods); it mandates intake of fish, chicken breast, and eggs [7]. EEN provides 100% of caloric intake via oral supplement and/or polymeric enteral support product (formula) [7]. The recommendation of whether to follow a dietary recommendation in children with IBD is made in consultation with the child’s healthcare team.

In addition, many caregivers of children with IBD turn to the internet for health information, including dietary guidance [9]. While several evidence-based dietary therapies for IBD are available, the quality of available internet information may be diminished by the recommendation of non-evidence-based dietary therapies. Internet dietary guidance may also be affected by readability, or the grade level required to understand the text. The readability of internet patient education is often poor, written above the recommended sixth to eighth grade level [10]. The primary objective of this study was to evaluate the quality and readability of internet dietary recommendations for pediatric IBD, including Crohn’s disease and ulcerative colitis. The secondary objective was to provide an overview of the dietary recommendations made on the internet for pediatric IBD.

## Materials and methods

DuckDuckGo and Startpage, two global non-trackable internet search engines that are not influenced by previous searches, were used to identify the top 30 results for each of the following searches: “IBD, diet, children”; “Crohn’s disease, diet, children”; and “ulcerative colitis, diet, children.” Websites that did not provide dietary information about IBD or were duplicates were excluded. Websites were classified into one of two pre-determined categories: (1) including dietary recommendations of any kind or (2) discussing diet only generally (e.g., diet as a risk factor for IBD) without mention of dietary modifications or specific diet regimens. The search was conducted in January 2025.

Two trainee reviewers independently rated each uniquely identified website using the DISCERN instrument, a tool for assessing consumer health information that was validated using a panel of experts on consumer health information and public health [11]. DISCERN contains 16 questions, each scored from one to five (or counted as a zero if the question is not applicable), and organized into categories including reliability, information quality, and overall quality. Reliability evaluates website aims, sources, and degree of bias. Information quality includes a description of risks and benefits, impact on quality of life, discussion of multiple treatment options, and support for shared decision-making. Overall quality is comprised of a single subjective score considering all components together. DISCERN includes “hints” to inform scoring for each question, with a focus on determining the presence or absence of key website components (e.g., “Is it clear what sources of information were used to compile the publication?” or “Is it clear that there may be more than one treatment choice?”) and their corresponding level of detail, such that reliable use of the tool does not require raters to be experts in the subject matter. Scores were classified as low (<3), moderate (≥3 to ≤4), or high (>4) [11]. The interrater reliability of the two independent reviewers was assessed using an intraclass correlation coefficient, with the following interpretation parameters: <0.50, poor; ≥0.50 to <0.75, moderate; ≥0.75 to <0.90, good; and ≥0.90, excellent. Means and standard deviations were calculated using Microsoft Excel (Microsoft Corp., Redmond, WA, US). Intraclass correlation coefficient analyses were performed using the online calculator StatsToDo [12].

The readability of each uniquely identified website was evaluated using the Flesch-Kincaid grade level (FKGL) measure, a standardized tool for determining the grade level at which text is written [13]. FKGL was determined using Microsoft Word (Microsoft Corp.).

During website review, reviewers captured each dietary recommendation provided and documented them using language consistent with that of the websites, and whether the state of IBD (e.g., stricture) was included in the recommendation. Specific dietary recommendations were subcategorized into the categories of “artificial nutrition,” “targeted dietary modifications,” and “structured named diets” following initial review of the recommendations made by the websites.

## Results

Of the 180 initially identified websites, 52 were excluded (five for irrelevant content and 47 for being duplicates). Of the 128 included websites, 46 met criteria for “IBD, diet, children”; 43 for “Crohn’s disease, diet, children”; and 39 for “ulcerative colitis, diet, children.” Inter-rater agreement was good to excellent (Table 1). Across all search terms, mean reliability was found to be moderate, while mean information quality and mean overall quality were found to be poor (Table 1). The mean FKGL grade level and standard deviation were 11.9 ± 3.0 for “IBD, diet, children”; 10.8 ± 2.4 for “Crohn’s disease, diet, children”; and 11.1 ± 2.8 for “ulcerative colitis, diet, children” (Table 1).

**Table 1:** Reliability, Information Quality, and Overall Quality of Internet Dietary Information for Pediatric Inflammatory Bowel Disease (IBD) using the DISCERN Instrument ^1^Classified as low (<3), moderate, (≥3 to ≤4), or high (>4) SD: standard deviation; ICC: intraclass correlation coefficient, classified as poor (<0.50), moderate (≥0.50 to <0.75), good (≥0.75 to <0.90), or excellent (≥0.90); CI: confidence interval; df: degrees of freedom

Search term and DISCERN categories	Mean^1^ (±SD)	ICC (95% CI)	F-ratio for ICC (df1, df2)	p-value for ICC
IBD, diet, children (n = 46)				
Reliability	3.1 ± 1.6	0.81 (0.77-0.84)	9.66 (367, 367)	<0.0001
Quality of information	2.5 ± 1.4	0.87 (0.84-0.89)	14.47 (321, 321)	<0.0001
Overall quality	2.8 ± 1.2	0.96 (0.92-0.98)	44.89 (45, 45)	<0.0001
Flesch-Kincaid grade level	11.9 ± 3.0	-	-	-
Crohn’s disease, diet, children (n = 43)				
Reliability	3.0 ± 1.6	0.80 (0.76-0.84)	9.37 (343, 343)	<0.0001
Quality of information	2.5 ± 1.4	0.87 (0.84-0.89)	14.05 (300, 300)	<0.0001
Overall quality	2.7 ± 1.0	0.92 (0.85-0.95)	23.63 (42, 42)	<0.0001
Flesch-Kincaid grade level	10.8 ± 2.4	-	-	-
Ulcerative colitis, diet, children (n = 39)				
Reliability	3.1 ± 1.6	0.78 (0.73-0.82)	8.11 (311, 311)	<0.0001
Quality of information	2.4 ± 1.4	0.86 (0.82-0.89)	13.56 (272, 272)	<0.0001
Overall quality	2.9 ± 1.1	0.91 (0.84-0.95)	21.27 (38, 38)	<0.0001
Flesch-Kincaid grade level	11.1 ± 2.8	-	-	-

For the IBD, Crohn’s disease, and ulcerative colitis searches, 39 (85%), 38 (88%), and 33 (85%) of websites, respectively, included specific dietary recommendations (Table 2). The remainder provided general dietary information alone (e.g., discussing diet as a risk factor for IBD). Included websites made 23 unique dietary recommendations, such as artificial nutrition (e.g., EEN), targeted dietary modifications (e.g., low red meat), and structured named diets (e.g., SCD) (Table 2). These recommendations were diverse and inconsistent, though the most common was for EEN in 50% of IBD and 51% of Crohn’s disease search results.

**Table 2:** Online Dietary Recommendations for Pediatric Inflammatory Bowel Disease (IBD) CDED: Crohn’s Disease Exclusion Diet; UCED: Ulcerative Colitis Exclusion Diet; FODMAP: fermentable oligosaccharide monosaccharide and polyols; CD-TREAT: Crohn’s Disease Treatment with Eating

Search term	IBD, diet, children (n = 46)	Crohn’s disease, diet, children (n = 43)	Ulcerative colitis, diet, children (n = 39)
Specific recommendations (as named by websites)	39 (85%)	38 (88%)	33 (85%)
Artificial nutrition			
Exclusive enteral	23 (50%)	22 (51%)	11 (28%)
Partial or unspecified enteral	12 (26%)	15 (35%)	8 (21%)
Parenteral	6 (13%)	3 (7%)	4 (10%)
Targeted dietary modifications			
Low lactose and/or dairy	7 (15%)	10 (23%)	9 (23%)
Low gas-producing foods	1 (2%)	4 (9%)	3 (8%)
Low-fat and/or fried foods	15 (33%)	12 (28%)	13 (33%)
Low gluten	1 (2%)	-	1 (3%)
Low red meat	4 (9%)	3 (7%)	3 (8%)
Avoid spicy foods	4 (9%)	5 (12%)	5 (13%)
Low fiber, nuts/seeds, and/or raw vegetables	12 (26%)	14 (33%)	15 (38%)
Low caffeine, alcohol, and/or artificial sweeteners	11 (24%)	13 (30%)	12 (31%)
Low processed foods, added sugar, and/or sorbitol	23 (50%)	12 (28%)	16 (41%)
High fiber	3 (7%)	2 (5%)	2 (5%)
High protein	1 (2%)	4 (9%)	4 (10%)
Structured named diets			
Specific carbohydrate diet	13 (28%)	4 (9%)	9 (23%)
CDED and/or UCED	11 (24%)	13 (30%)	10 (26%)
Anti-inflammatory diet	5 (11%)	2 (5%)	2 (5%)
Mediterranean diet	4 (9%)	2 (5%)	5 (13%)
Low FODMAP	4 (9%)	2 (5%)	5 (13%)
CD-TREAT	2 (4%)	3 (7%)	2 (5%)
Semi-vegetarian	2 (4%)	1 (2%)	1 (3%)
Autoimmune protocol	2 (4%)	1 (2%)	2 (5%)
Zinc-enriched Japanese diet	-	-	1 (3%)
General information only	7 (15%)	5 (12%)	6 (15%)
Refer to provider	3 (7%)	5 (12%)	5 (13%)
Diet as a risk factor for IBD	4 (9%)	-	1 (3%)

Targeted dietary recommendations lacked disease state-tailored contextualization in 52%, 41%, and 37% of relevant IBD, Crohn’s disease, and ulcerative colitis websites, respectively. Diets low in fiber, nuts/seeds, and/or raw vegetables were the most contextualized recommendations among the IBD, Crohn’s disease, and ulcerative colitis websites, with 75%, 64%, and 80%, respectively, specifying relevance during flare and/or stricturing disease. Conversely, recommendations of diets low in gas-producing foods and low gluten were never contextualized.

## Discussion

The internet is a leading source of health information for patients and the general public, offering rapid access to a multitude of resources [14]. However, the quality of online health information from over 11,000 websites evaluated prior to and including 2017 has generally been found to be suboptimal [14]. Even if the quality of health information is excellent, readability, which assesses how easily written material can be read and comprehended by the intended audience, affects the usage of the information. Unfortunately, we identified a reliability, quality, and readability crisis in internet dietary information for pediatric IBD, where mean information quality and mean overall quality were found to be poor, and readability was at an excessively high reading level.

There was a high variance in recommendations, with more than 20 recommended dietary changes. Many recommendations lacked clinical contextualization related to the IBD disease state such as flare versus remission or disease features such as strictures. Based on our findings, caregivers and children with IBD following dietary internet advice may be at increased risk for exposure to low-quality information with varied dietary recommendations, including unproven restrictive diets. This is concerning, given the unique developmental and nutritional needs of children with IBD [15].

The identification of poor-quality information from reviewed websites for pediatric IBD searches was disappointing. Nonetheless, our findings parallel a recent Chinese language evaluation of adult IBD-related information, which identified moderate to low-quality information [16]. Therefore, our study specifically demonstrates that children with IBD are also susceptible to the consequences of this information quality crisis. Poor quality information can have significant consequences for health, as most caregivers search online for health information when their child is ill, with many following internet advice instead of visiting a doctor [17]. One study found that patients with IBD receive nearly as much information from the internet as from their gastroenterologist [18]. Our findings highlight the opportunity for providers of children with IBD to discuss potential issues with the quality of internet dietary information and, ideally, provide high-quality dietary recommendations in their practice.

Additionally, readability seemed excessively difficult for the websites reviewed. The average reading level of adults in the United States is eighth grade, which is well below our findings of website text being written at an average reading level of 11th grade [19]. Readability can be readily assessed using validated tools and formulas. Future internet health information efforts in this area can consider using the FKGL instrument, as was used in this study, or take advantage of online plain language tools that are being developed [20]. Ultimately, the use of plain language with the avoidance of technical terms and providing an engaging information structure may help improve readability and comprehension [20].

The high number (>20) of unique dietary recommendations contributed to the high variation identified in the study. While a few dietary recommendations have evidence of efficacy in children with IBD, such as EEN, or a modified CDED, many have either only evidence in adults with IBD or no evidence of efficacy for those with IBD at all [21]. Therefore, there appear to be opportunities for websites to highlight those nutritional recommendations with published efficacy data (particularly for Crohn’s disease) versus those that do not, including explicit acknowledgement of uncertainty when relevant in recommending dietary change.

Ultimately, high-quality and more easily readable pediatric IBD dietary resources are needed online. For now, providers may consider reviewing and directing patients to specific websites. In our analysis, internet dietary information from the Crohn’s & Colitis Foundation had the highest composite DISCERN scores, suggesting their potential as a quality internet resource. However, the text for these websites was still written at an approximately 11th-grade reading level or higher, suggesting future opportunities to address readability. An increased focus on multidisciplinary care should additionally be considered, as research has shown that engagement of dieticians improves patient outcomes in IBD [22].

Strengths of this study include being the first study evaluating internet dietary information for searches specific to pediatric IBD. Additionally, the study used validated instruments to evaluate information reliability, quality, and readability. Two independent raters were used, with high inter-rater reliability.

Study limitations include its focus on internet search results, while many patients, particularly of younger age, are turning to social media for health advice [23]. Future research may evaluate IBD dietary information on these platforms. Our focus on English-language results also limits generalizability, particularly when considering patients whose primary language is not English. We also recognize that, as with any subjective evaluation, there is the possibility of implicit biases influencing reviewer DISCERN scoring.

We chose to use the search engines DuckDuckGo and Startpage to prioritize standardization and reproducibility of search results as their output are not influenced by user behavior (e.g., previous searches). However, a limitation of this study is that patient experiences may vary with the use of more commonly used alternative search engines, which produce variable results between users. Additionally, results may differ based on inevitable differences in search term phrasing between patients. We designed the search terms utilized in this study to include the most pertinent keywords likely to be consistent across most patient searches relevant to pediatric IBD dietary information. Our approach also limits analyses to the top search results at the time of data collection, potentially missing higher-quality yet less popular websites. It is possible that top search results could fluctuate over time or evolve as new websites are published.

## Conclusions

The mean reliability of current internet dietary information for pediatric IBD, including an evaluation of website sources and bias, was found to be moderate. The mean quality of the information conveyed about dietary management of pediatric IBD, including risks, benefits, and treatment options, was found to be low. The mean overall quality rating of websites about diet and pediatric IBD was also found to be low. Furthermore, these websites were presented at a high reading complexity, with a mean 11th-grade reading level, and dietary recommendations were numerous and inconsistent across websites. Providers for children with IBD may consider counseling families on the challenges of seeking internet dietary information and, ideally, discuss dietary recommendations in their practice, including multidisciplinary nutritional support and with close follow-up.

## Appendices

Appendix A

**Table 3:** DISCERN Scores for “IBD, diet, children” by Question (Q) and Reviewer (R)

	Reliability																Information quality														Overall quality	
Website number	Q1 R1	Q1 R2	Q2 R1	Q2 R2	Q3 R1	Q3 R2	Q4 R1	Q4 R2	Q5 R1	Q5 R2	Q6 R1	Q6 R2	Q7 R1	Q7 R2	Q8 R1	Q8 R2	Q1 R1	Q1 R2	Q2 R1	Q2 R2	Q3 R1	Q3 R2	Q4 R1	Q4 R2	Q5 R1	Q5 R2	Q6 R1	Q6 R2	Q7 R1	Q7 R2	R1	R2
1	3	3	5	4	5	5	3	3	3	1	4	4	5	5	2	2	2	3	2	3	1	1	1	1	3	3	3	3	2	3	4	4
2	1	1	0	0	5	4	1	1	3	1	3	4	3	3	1	1	2	2	2	3	2	1	2	1	2	2	3	4	1	1	3	3
3	4	3	4	5	1	1	5	5	1	1	3	3	1	1	1	1	2	2	3	3	1	1	3	3	2	3	1	1	1	1	1	1
4	1	1	0	0	5	5	1	3	2	5	4	3	1	1	2	1	5	5	5	5	1	1	3	3	2	2	5	5	2	2	4	4
5	2	2	4	5	4	4	3	1	3	1	4	3	4	5	2	2	2	3	4	5	1	1	1	3	4	3	5	5	4	2	4	4
6	5	5	5	5	5	5	5	5	3	3	4	4	4	5	5	5	4	3	5	5	4	3	1	1	3	3	5	5	3	3	5	5
7	2	3	1	3	1	1	4	3	3	1	2	2	1	1	1	1	1	2	3	3	1	1	2	1	1	3	3	3	3	5	1	1
8	1	1	0	0	3	3	3	3	3	3	4	4	3	3	2	2	2	2	4	3	1	1	5	4	5	4	3	3	3	3	3	3
9	1	1	0	0	1	2	3	2	2	1	4	4	3	3	3	4	1	1	3	3	1	1	1	1	2	2	3	3	3	3	2	2
10	5	5	5	5	2	3	5	5	5	5	5	5	1	3	3	3	1	1	3	3	1	1	3	3	2	2	5	5	2	2	2	3
11	5	5	5	5	5	5	5	5	5	5	5	5	1	3	3	3	3	3	5	5	5	5	5	5	3	3	5	5	1	2	5	5
12	5	5	5	5	2	2	5	5	5	5	5	5	1	3	5	5	2	2	3	3	1	1	1	1	3	2	5	5	3	3	3	3
13	1	1	0	0	3	2	3	1	3	1	4	4	4	3	2	3	2	2	3	3	1	1	1	1	3	3	5	5	4	3	2	2
14	3	3	3	3	2	2	3	1	3	1	3	4	3	5	1	2	1	1	1	2	1	1	5	2	1	1	5	5	1	1	2	2
15	1	1	0	0	3	2	3	1	3	3	4	3	3	5	3	3	2	2	1	1	1	1	5	3	1	1	5	5	4	4	3	3
16	3	3	3	3	3	3	5	5	3	3	3	3	3	5	5	5	3	3	3	3	1	1	1	1	1	1	4	3	1	2	3	3
17	2	3	3	3	4	4	5	5	5	3	4	4	3	3	3	3	3	3	3	3	2	3	1	2	2	2	2	3	3	3	3	3
18	2	3	5	5	5	5	5	5	5	3	5	5	5	5	5	5	5	3	5	3	5	3	1	1	5	3	5	5	3	3	5	4
19	1	1	0	0	3	3	2	3	3	3	4	4	5	5	1	1	5	3	5	3	1	1	2	1	1	3	5	5	3	3	2	2
20	5	5	3	5	2	3	5	5	5	5	5	5	1	1	5	5	2	3	2	3	4	3	2	2	2	2	5	5	1	1	2	3
21	4	5	4	5	3	3	5	5	5	5	5	5	1	1	3	3	2	2	4	3	2	3	2	1	3	3	5	5	1	1	4	4
22	1	1	0	0	1	1	3	3	2	3	3	3	4	5	1	3	1	1	1	1	1	1	1	1	2	3	1	1	1	1	1	1
23	2	3	4	3	2	2	5	5	5	5	4	3	1	5	1	2	4	5	3	3	1	1	1	1	2	1	1	1	2	2	1	2
24	1	1	0	0	4	3	3	3	3	1	4	4	3	3	4	4	2	2	2	2	1	1	4	3	4	3	5	5	5	5	4	4
25	5	5	3	5	3	5	3	3	3	3	3	4	5	5	5	5	3	5	3	3	1	1	1	1	2	2	5	5	4	3	4	4
26	1	1	0	0	4	3	3	3	3	1	4	4	3	3	4	4	2	2	3	2	1	1	4	3	4	3	5	5	5	5	4	4
27	5	5	5	5	5	5	5	5	5	5	4	5	4	5	2	2	5	5	3	3	2	2	5	3	5	5	5	5	5	5	5	5
28	5	5	5	5	1	2	5	5	5	5	3	3	1	3	2	3	1	1	1	1	1	1	1	1	1	1	1	1	1	1	1	1
29	1	1	0	0	4	3	5	4	3	1	5	3	1	1	3	2	2	2	2	2	3	2	1	1	2	2	5	5	2	2	3	3
30	1	1	0	0	3	3	3	3	3	1	3	4	1	3	2	2	2	2	2	2	1	1	1	2	2	3	2	3	2	2	2	2
31	5	5	3	5	3	3	5	5	5	5	5	5	1	1	2	3	4	3	4	4	4	3	1	1	3	3	5	5	1	1	3	3
32	4	3	4	3	3	3	4	5	3	5	4	5	4	5	2	2	3	3	2	2	1	1	1	1	1	1	4	3	3	3	3	3
33	1	1	0	0	3	3	3	2	2	1	3	3	3	3	1	1	5	3	5	5	1	1	3	3	2	3	1	1	3	3	2	2
34	2	2	2	2	2	2	3	1	3	1	4	4	1	3	2	1	4	3	3	3	1	1	1	1	2	1	4	3	3	2	3	2
35	1	1	0	0	2	2	1	5	2	5	4	4	3	3	2	3	2	2	3	2	1	1	1	1	2	1	5	3	3	3	2	2
36	1	1	0	0	2	2	2	3	3	3	3	3	4	3	3	3	2	2	4	1	1	1	1	1	3	1	1	1	1	1	1	1
37	2	2	2	2	2	2	3	1	3	1	3	4	1	1	2	3	1	1	2	3	2	1	1	1	1	1	1	3	3	3	2	2
38	1	1	0	0	2	2	3	3	3	3	3	3	5	5	1	3	5	5	5	5	5	5	1	1	3	3	4	3	3	3	3	3
39	5	5	5	5	3	4	5	5	5	5	5	5	1	1	5	5	1	1	5	5	1	1	1	1	3	3	5	5	1	1	3	4
40	5	3	3	3	3	3	5	5	3	3	5	4	5	5	2	3	3	3	3	3	1	1	1	1	1	1	5	5	1	3	3	3
41	5	5	5	5	5	5	5	5	5	5	5	5	1	1	5	5	5	5	5	5	3	3	5	3	2	2	5	5	1	1	5	5
42	3	3	3	5	3	3	4	3	3	3	4	3	4	5	1	2	3	3	3	3	2	3	1	1	2	2	4	5	4	5	3	3
43	1	3	0	3	1	2	5	5	5	5	2	3	1	5	1	3	1	1	1	1	1	1	1	1	1	1	1	1	1	1	1	1
44	5	5	5	5	1	2	1	3	4	3	4	4	3	5	4	5	1	1	1	1	1	1	1	1	1	1	1	1	1	1	1	1
45	3	3	3	3	1	2	5	5	4	5	3	4	1	1	1	3	1	1	1	1	1	1	1	1	1	1	1	1	1	1	1	1
46	1	1	0	0	3	3	3	3	3	1	4	4	1	1	5	5	1	2	2	2	1	1	1	3	2	2	5	3	2	2	3	3

Websites for “IBD, diet, children”:

1. https://www.crohnscolitisfoundation.org/youth-parent-resources/diet-and-nutrition

2. https://www.stlouischildrens.org/conditions-treatments/inflammatory-bowel-disease-ibd-center/education/diet-inflammatory-bowel-disease

3. https://www.hopkinsmedicine.org/health/conditions-and-diseases/inflammatory-bowel-disease/ibd-in-children-answers-from-a-pediatric-gastroenterologist-maria-oliva-hemker

4. https://www.massgeneral.org/children/inflammatory-bowel-disease/different-diets-for-ibd

5. https://www.stanfordchildrens.org/en/services/inflammatory-bowel-disease/services/ibd-nutritional-therapy

6. https://www.luriechildrens.org/en/blog/common-questions-about-dietary-therapy-for-ibd/

7. https://crohnsandcolitisdietitians.com/ibd-in-kids/

8. https://www.cincinnatichildrens.org/health/i/ibd

9. https://www.childrenshospital.org/conditions/inflammatory-bowel-disease

10. https://www.espen.org/files/ESPEN-Guidelines/ESPEN_guideline_on_Clinical_nutrition_in_inflammatory_bowel_disease.pdf

11. https://pmc.ncbi.nlm.nih.gov/articles/PMC7922138/

12. https://publications.aap.org/pediatrics/article/151/1/e2022058037/190342/Pediatric-Inflammatory-Bowel-Disease?autologincheck=redirected

13. https://www.childrensnational.org/get-care/health-library/ulcerative-colitis-in-children

14. https://www.childrensdayton.org/understanding-childrens-inflammatory-bowel-disease

15. https://www.seattlechildrens.org/clinics/gastroenterology-hepatology/treatments-and-services/ibd/

16. https://www.health.harvard.edu/blog/i-have-inflammatory-bowel-disease-ibd-what-should-i-eat-2020051819799

17. https://www.crohnscolitisfoundation.org/patientsandcaregivers/diet-and-nutrition/what-should-i-eat

18. https://www.crohnscolitisfoundation.org/patientandcaregivers/diet-and-nutrition/special-ibd-diets

19. https://www.rch.org.au/kidsinfo/fact_sheets/Inflammatory_bowel_disease/

20. https://pmc.ncbi.nlm.nih.gov/articles/PMC4021001/

21. https://www.gastrojournal.org/article/S0016-5085(23)05597-X/fulltext

22. https://medicalxpress.com/news/2024-10-inflammatory-bowel-disease-crucial-period.html

23. https://www.dietvsdisease.org/diy-low-fodmap-diet/

24. https://www.seattlechildrens.org/conditions/crohns-disease/

25. https://crohnsandcolitis.org.uk/info-support/information-about-crohns-and-colitis/all-information-about-crohns-and-colitis/living-with-crohns-or-colitis/food

26. https://www.seattlechildrens.org/conditions/ulcerative-colitis/

27. https://www.crohnscolitisfoundation.org/sites/default/files/2020-03/diet-and-nutrition-brochure.pdf

28. https://pmc.ncbi.nlm.nih.gov/articles/PMC10958293/

29. https://www.cicra.org/what-is-ibd/diet-and-nutrition/

30. https://gikids.org/resource/ibd-general-nutrition-information/

31. https://www.sciencedirect.com/science/article/pii/S2667009724000241

32. https://www.healthline.com/health/ulcerative-colitis/pediatric-ulcerative-colitis-diet

33. https://www.childrenshospital.org/treatments/een

34. https://nyulangone.org/conditions/crohns-disease-in-children/treatments/nutrition-for-crohns-disease-in-children

35. https://www.webmd.com/ibd-crohns-disease/ulcerative-colitis/ss/slideshow-uc-child-nutrition

36. https://www.seattlechildrens.org/healthcare-professionals/provider-news/treating-ibd-diet/

37. https://www.uhhospitals.org/services/digestive-health-services/conditions-and-treatments/small-and-large-intestine/inflammatory-bowel-disease/diet-guide

38. https://kidshealth.org/en/parents/nutrition-crohns.html

39. https://pmc.ncbi.nlm.nih.gov/articles/PMC4924175/

40. https://blog.ochsner.org/articles/how-can-nutrition-therapy-help-kids-with-ibd

41. https://www.liebertpub.com/doi/full/10.1089/jmf.2019.0063

42. https://www.mhealthfairview.org/blog/five-things-to-know-nutrition-therapy-for-pediatric-crohns-disease

43. https://www.news-medical.net/news/20240205/High-quality-early-diet-linked-to-lower-IBD-risk-in-children.aspx

44. https://www.gu.se/en/news/healthy-diet-early-in-life-seems-to-protect-against-inflammatory-bowel-disease

45. https://academic.oup.com/ibdjournal/article/14/3/367/4653615

46. https://gikids.org/resource/ibd-nutrition-guide

Appendix B

**Table 4:** DISCERN Scores for “Crohn’s disease, diet, children” by Question (Q) and Reviewer (R)

	Reliability																Information quality														Overall quality	
Website number	Q1 R1	Q1 R2	Q2 R1	Q2 R2	Q3 R1	Q3 R2	Q4 R1	Q4 R2	Q5 R1	Q5 R2	Q6 R1	Q6 R2	Q7 R1	Q7 R2	Q8 R1	Q8 R2	Q1 R1	Q1 R2	Q2 R1	Q2 R2	Q3 R1	Q3 R2	Q4 R1	Q4 R2	Q5 R1	Q5 R2	Q6 R1	Q6 R2	Q7 R1	Q7 R2	R1	R2
1	3	3	5	4	5	5	3	3	3	1	4	4	5	5	2	2	2	3	2	3	1	1	1	1	3	3	3	3	2	3	4	4
2	1	1	0	0	5	4	1	1	3	1	3	4	3	3	1	1	2	2	2	3	2	1	2	1	2	2	3	4	1	1	3	3
3	2	3	3	3	4	4	5	5	5	3	4	4	3	3	3	3	3	3	3	3	2	3	1	2	2	2	2	3	3	3	3	3
4	2	3	5	5	5	5	5	5	5	3	5	5	5	5	5	5	5	3	5	3	5	3	1	1	5	3	5	5	3	3	5	4
5	1	1	0	0	3	3	2	3	3	3	4	4	5	5	1	1	5	3	5	3	1	1	2	1	1	3	5	5	3	3	2	2
6	1	1	0	0	4	3	3	3	3	1	4	4	3	3	4	4	2	2	2	2	1	1	4	3	4	3	5	5	5	5	4	4
7	5	5	5	5	5	5	5	5	5	5	4	5	4	5	2	2	5	5	3	3	2	2	5	3	5	5	5	5	5	5	5	5
8	1	1	0	0	4	3	5	4	3	1	5	3	1	1	3	2	2	2	2	2	3	2	1	1	2	2	5	5	2	2	3	3
9	1	1	0	0	3	3	3	3	3	1	3	4	1	3	2	2	2	2	2	2	1	1	1	2	2	3	2	3	2	2	2	2
10	5	5	3	5	3	3	5	5	5	5	5	5	1	1	2	3	4	3	4	4	4	3	1	1	3	3	5	5	1	1	3	3
11	1	1	0	0	3	3	3	2	2	1	3	3	3	3	1	1	5	3	5	5	1	1	3	3	2	3	1	1	3	3	2	2
12	2	2	2	2	2	2	3	1	3	1	4	4	1	3	2	1	4	3	3	3	1	1	1	1	2	1	4	3	3	2	3	2
13	1	1	0	0	2	2	1	5	2	5	4	4	3	3	2	3	2	2	3	2	1	1	1	1	2	1	5	3	3	3	2	2
14	1	1	0	0	2	2	3	3	3	3	3	3	5	5	1	3	5	5	5	5	5	5	1	1	3	3	4	3	3	3	3	3
15	5	3	3	3	3	3	5	5	3	3	5	4	5	5	2	3	3	3	3	3	1	1	1	1	1	1	5	5	1	3	3	3
16	3	3	3	5	3	3	4	3	3	3	4	3	4	5	1	2	3	3	3	3	2	3	1	1	2	2	4	5	4	5	3	3
17	1	1	0	0	3	3	3	1	3	1	4	4	2	1	3	3	3	3	3	3	1	1	5	5	2	3	3	3	4	5	4	4
18	4	5	5	5	2	3	5	5	3	5	5	5	3	5	2	3	2	2	3	2	1	1	2	2	5	5	2	3	5	5	2	2
19	1	1	0	0	5	5	1	5	2	5	4	5	3	5	2	3	4	4	4	4	1	1	2	2	2	2	5	5	3	3	4	4
20	1	1	0	0	2	2	5	3	5	5	3	3	4	3	3	3	1	1	2	2	1	1	3	2	1	1	2	2	1	1	2	2
21	1	1	0	0	2	2	3	1	3	1	4	4	3	5	1	1	1	2	2	2	1	1	1	1	1	1	1	2	1	2	1	1
22	1	1	0	0	2	2	3	5	3	5	4	5	5	5	2	2	2	2	3	2	1	1	1	1	1	1	4	3	1	1	2	2
23	1	1	0	0	3	3	2	5	3	5	3	4	3	5	2	3	5	5	5	5	5	5	1	1	3	3	1	1	2	2	2	3
24	2	3	5	5	5	4	5	5	5	5	5	5	3	3	2	3	3	3	4	3	2	2	1	1	2	1	5	5	3	3	3	3
25	1	1	0	0	2	2	3	1	3	1	3	4	3	3	2	3	2	1	1	1	1	1	1	1	1	1	1	1	1	3	1	1
26	5	5	5	5	2	2	5	5	5	5	3	4	1	1	5	5	5	5	5	5	2	3	1	1	2	2	2	3	1	1	2	2
27	1	1	0	0	2	3	3	3	3	3	3	4	3	5	1	1	4	3	2	2	1	1	1	1	2	1	2	1	2	2	2	2
28	5	5	5	5	5	5	5	5	5	5	5	5	5	5	5	5	3	3	4	4	2	2	1	1	2	2	5	5	3	3	4	5
29	5	5	3	3	2	3	5	5	5	5	3	2	3	3	2	3	5	3	3	3	1	1	1	1	2	2	4	3	3	3	2	2
30	1	1	0	0	3	3	3	3	3	1	4	4	1	3	3	3	3	2	3	3	1	1	5	5	2	2	3	3	4	5	4	4
31	1	1	0	0	2	2	2	5	3	5	4	4	1	3	2	3	1	1	1	1	1	1	1	1	1	1	2	2	1	1	1	1
32	5	5	5	5	3	3	5	5	5	5	5	5	1	1	5	5	4	5	5	5	1	1	1	1	2	1	5	5	1	1	3	3
33	4	5	5	5	3	3	5	5	5	5	5	5	1	1	5	5	3	3	4	3	1	1	2	2	2	2	5	5	1	1	3	3
34	5	5	5	5	2	2	5	5	5	5	3	5	2	3	1	1	3	3	3	3	1	1	2	3	1	1	2	3	2	3	2	3
35	1	1	0	0	2	2	3	3	3	3	3	3	1	3	3	3	5	3	5	3	1	1	1	1	2	1	1	1	2	2	2	2
36	5	5	4	5	2	2	5	5	5	5	4	4	1	1	5	5	3	5	3	3	1	1	1	1	2	2	4	3	1	1	2	3
37	1	1	0	0	2	2	1	3	3	3	3	3	1	1	2	5	2	5	3	3	1	1	1	1	1	1	1	1	1	1	1	2
38	5	5	5	5	4	2	5	5	5	5	4	4	1	1	5	5	4	5	5	3	4	5	1	1	5	5	2	3	4	3	3	3
39	1	1	0	0	2	2	3	1	3	3	3	3	4	5	2	2	1	1	1	1	1	1	1	1	1	1	4	3	2	2	2	2
40	1	2	0	3	3	3	3	3	2	1	3	4	3	3	3	3	2	2	2	3	1	1	3	3	2	1	2	3	2	3	3	3
41	5	5	4	5	3	3	5	5	4	3	5	5	1	5	3	3	4	3	3	3	3	3	5	5	2	1	5	5	1	1	4	4
42	1	1	0	0	2	2	3	1	3	1	3	3	3	5	1	3	1	1	3	1	1	1	1	1	1	1	1	1	3	3	2	2
43	1	1	0	0	2	2	3	1	3	1	4	4	1	3	1	2	2	2	2	2	1	1	2	1	1	1	4	3	2	3	2	2

Websites for “Crohn’s disease, diet, children”:

1. https://www.crohnscolitisfoundation.org/youth-parent-resources/diet-and-nutrition

2. https://www.stlouischildrens.org/conditions-treatments/inflammatory-bowel-disease-ibd-center/education/diet-inflammatory-bowel-disease

3. https://www.crohnscolitisfoundation.org/patientsandcaregivers/diet-and-nutrition/what-should-i-eat

4. https://www.crohnscolitisfoundation.org/patientandcaregivers/diet-and-nutrition/special-ibd-diets

5. https://www.rch.org.au/kidsinfo/fact_sheets/Inflammatory_bowel_disease/

6. https://www.seattlechildrens.org/conditions/crohns-disease/

7. https://www.crohnscolitisfoundation.org/sites/default/files/2020-03/diet-and-nutrition-brochure.pdf

8. https://www.cicra.org/what-is-ibd/diet-and-nutrition/

9. https://gikids.org/resource/ibd-general-nutrition-information/

10. https://www.sciencedirect.com/science/article/pii/S2667009724000241

11. https://www.childrenshospital.org/treatments/een

12. https://nyulangone.org/conditions/crohns-disease-in-children/treatments/nutrition-for-crohns-disease-in-children

13. https://www.webmd.com/ibd-crohns-disease/ulcerative-colitis/ss/slideshow-uc-child-nutrition

14. https://kidshealth.org/en/parents/nutrition-crohns.html

15. https://blog.ochsner.org/articles/how-can-nutrition-therapy-help-kids-with-ibd

16. https://www.mhealthfairview.org/blog/five-things-to-know-nutrition-therapy-for-pediatric-crohns-disease

17. https://www.stanfordchildrens.org/en/topic/default?id=crohns-disease-in-children-90-P01987

18. https://www.mycrohnsandcolitisteam.com/resources/diet-for-child-with-crohns-disease-6-tips-for-nutrition

19. https://www.webmd.com/ibd-crohns-disease/crohns-disease/creating-a-crohns-disease-diet-plan

20. https://www.niddk.nih.gov/health-information/digestive-diseases/crohns-disease/eating-diet-nutrition

21. https://www.yalemedicine.org/conditions/pediatric-crohns-disease

22. https://www.mycrohnsandcolitisteam.com/resources/crohns-disease-in-children-symptoms-causes-treatment

23. https://www.healthline.com/health/crohns-disease-exclusion-diet

24. https://www.gastroenterologyadvisor.com/features/ibd-diet/

25. https://www.chop.edu/conditions-diseases/crohns-disease

26. https://pmc.ncbi.nlm.nih.gov/articles/PMC10650058/

27. https://www.nutritionaltherapyforibd.org/dietary-options/crohns-disease-exclusion-diet

28. https://www.crohnscolitisfoundation.org/sites/default/files/legacy/assets/pdfs/diet-nutrition-2013.pdf

29. https://crohnsandcolitisdietitians.com/crohns-disease-exclusion-diet/

30. https://www.urmc.rochester.edu/encyclopedia/content?ContentTypeID=90&ContentID=P01987

31. https://www.webmd.com/ibd-crohns-disease/crohns-disease/crohns-disease-in-children-and-teens

32. https://pmc.ncbi.nlm.nih.gov/articles/PMC8150738/

33. https://pmc.ncbi.nlm.nih.gov/articles/PMC7828385/

34. https://www.mountsinai.org/health-library/discharge-instructions/crohn-disease-children-discharge

35. https://www.albertahealthservices.ca/assets/info/nutrition/if-nfs-nutrition-therapy-children-with-crohns-disease.pdf

36. https://www.tandfonline.com/doi/full/10.1080/03007995.2021.1920901

37. https://imperialbrc.nihr.ac.uk/2020/11/12/diet-for-crohns/

38. https://pghn.org/DOIx.php?id=10.5223/pghn.2023.26.3.165

39. https://www.nhs.uk/conditions/crohns-disease/living-with/

40. https://www.ucsfhealth.org/education/nutrition-tips-for-inflammatory-bowel-disease

41. https://www.choc.org/wp-content/uploads/2017/02/2-RypkemaL-RDsInPracticeGI.pdf

42. https://www.childrenscolorado.org/conditions-and-advice/conditions-and-symptoms/conditions/crohns-disease/

43. https://www.columbiadoctors.org/childrens-health/pediatric-specialties/digestive-liver-disorders/treatments-conditions/crohns-disease

Appendix C

**Table 5:** DISCERN Scores for “Ulcerative colitis, diet, children” by Question (Q) and Reviewer (R)

	Reliability																Information quality														Overall quality	
Website number	Q1 R1	Q1 R2	Q2 R1	Q2 R2	Q3 R1	Q3 R2	Q4 R1	Q4 R2	Q5 R1	Q5 R2	Q6 R1	Q6 R2	Q7 R1	Q7 R2	Q8 R1	Q8 R2	Q1 R1	Q1 R2	Q2 R1	Q2 R2	Q3 R1	Q3 R2	Q4 R1	Q4 R2	Q5 R1	Q5 R2	Q6 R1	Q6 R2	Q7 R1	Q7 R2	R1	R2
1	3	3	5	4	5	5	3	3	3	1	4	4	5	5	2	2	2	3	2	3	1	1	1	1	3	3	3	3	2	3	4	4
2	1	1	0	0	5	4	1	1	3	1	3	4	3	3	1	1	2	2	2	3	2	1	2	1	2	2	3	4	1	1	3	3
3	1	1	0	0	5	5	1	3	2	5	4	3	1	1	2	1	5	5	5	5	1	1	3	3	2	2	5	5	2	2	4	4
4	5	5	5	5	5	5	5	5	5	5	5	5	1	3	3	3	3	3	5	5	5	5	5	5	3	3	5	5	1	2	5	5
5	1	1	0	0	3	2	3	1	3	1	4	4	4	3	2	3	2	2	3	3	1	1	1	1	3	3	5	5	4	3	2	2
6	2	3	3	3	4	4	5	5	5	3	4	4	3	3	3	3	3	3	3	3	2	3	1	2	2	2	2	3	3	3	3	3
7	2	3	5	5	5	5	5	5	5	3	5	5	5	5	5	5	5	3	5	3	5	3	1	1	5	3	5	5	3	3	5	4
8	1	1	0	0	3	3	2	3	3	3	4	4	5	5	1	1	5	3	5	3	1	1	2	1	1	3	5	5	3	3	2	2
9	1	1	0	0	4	3	3	3	3	1	4	4	3	3	4	4	2	2	3	2	1	1	4	3	4	3	5	5	5	5	4	4
10	5	5	5	5	5	5	5	5	5	5	4	5	4	5	2	2	5	5	3	3	2	2	5	3	5	5	5	5	5	5	5	5
11	1	1	0	0	4	3	5	4	3	1	5	3	1	1	3	2	2	2	2	2	3	2	1	1	2	2	5	5	2	2	3	3
12	5	5	3	5	3	3	5	5	5	5	5	5	1	1	2	3	4	3	4	4	4	3	1	1	3	3	5	5	1	1	3	3
13	4	3	4	3	3	3	4	5	3	5	4	5	4	5	2	2	3	3	2	2	1	1	1	1	1	1	4	3	3	3	3	3
14	1	1	0	0	2	2	1	5	2	5	4	4	3	3	2	3	2	2	3	2	1	1	1	1	2	1	5	3	3	3	2	2
15	1	1	0	0	2	2	2	3	3	3	3	3	4	3	3	3	2	2	4	1	1	1	1	1	3	1	1	1	1	1	1	1
16	2	2	2	2	2	2	3	1	3	1	3	4	1	1	2	3	1	1	2	3	2	1	1	1	1	1	1	3	3	3	2	2
17	1	3	0	3	1	2	5	5	5	5	2	3	1	5	1	3	1	1	1	1	1	1	1	1	1	1	1	1	1	1	1	1
18	2	3	5	5	5	4	5	5	5	5	5	5	3	3	2	3	3	3	4	3	2	2	1	1	2	1	5	5	3	3	3	3
19	5	5	5	5	5	5	5	5	5	5	5	5	5	5	5	5	3	3	4	4	2	2	1	1	2	2	5	5	3	3	4	5
20	1	2	0	3	3	3	3	3	2	1	3	4	3	3	3	3	2	2	2	3	1	1	3	3	2	1	2	3	2	3	3	3
21	5	5	4	5	3	3	5	5	4	3	5	5	1	5	3	3	4	3	3	3	3	3	5	5	2	1	5	5	1	1	4	4
22	1	1	0	0	2	2	4	5	3	5	2	3	3	5	2	3	1	1	1	1	1	1	1	1	2	3	3	3	4	3	2	2
23	2	3	3	5	3	4	5	5	5	5	5	5	3	5	2	3	1	1	2	3	2	3	1	1	2	1	3	3	3	3	2	4
24	1	1	0	0	2	2	3	1	3	3	3	3	3	3	3	3	1	1	2	2	1	1	2	2	1	1	2	1	2	2	2	1
25	1	1	0	0	2	2	3	1	3	1	4	4	1	5	1	3	3	2	2	2	1	1	2	1	1	1	4	3	2	3	2	2
26	1	1	0	0	2	2	3	1	3	1	3	3	1	1	1	3	1	1	1	1	1	1	1	1	1	1	1	1	1	1	1	1
27	1	1	0	0	3	2	5	5	5	5	5	5	3	3	4	3	1	1	1	1	1	1	4	3	1	1	5	5	2	3	3	3
28	1	1	0	0	5	5	3	1	3	3	4	4	3	5	4	3	5	5	3	3	2	2	1	1	3	5	5	5	2	3	4	4
29	2	1	2	0	3	3	5	5	5	5	5	5	1	3	4	3	3	2	3	2	1	1	2	2	2	2	5	5	3	5	4	4
30	5	5	5	5	3	3	5	5	5	5	5	5	1	1	5	5	3	3	4	3	1	1	2	3	2	2	5	5	1	1	3	3
31	1	1	0	0	1	1	5	5	5	5	4	4	1	5	2	3	1	1	1	1	2	1	2	1	1	1	1	2	2	2	1	1
32	1	1	0	0	3	3	5	5	5	5	4	4	3	5	2	3	2	2	2	1	1	1	2	1	2	1	2	2	1	2	2	2
33	1	1	0	0	2	2	5	5	5	5	4	4	2	3	2	3	1	1	1	1	2	1	2	1	1	1	2	3	2	2	2	2
34	5	5	5	5	4	3	3	5	3	5	5	5	5	5	4	3	2	1	3	1	2	1	1	1	1	1	3	3	2	2	3	2
35	4	5	4	5	3	2	3	1	2	1	4	3	3	3	3	3	1	1	3	3	2	1	1	1	1	1	4	4	3	3	3	3
36	1	1	0	0	2	2	3	1	3	1	3	3	3	3	5	3	1	1	4	3	1	1	3	3	2	1	2	3	3	3	3	3
37	2	1	5	0	5	5	5	5	5	5	5	5	5	5	5	3	4	3	3	3	1	1	1	1	2	1	5	5	3	3	5	4
38	1	1	0	0	5	5	4	3	3	3	4	4	2	3	2	3	1	3	5	5	1	1	1	1	2	2	4	3	5	5	3	3
39	5	5	5	5	2	3	5	5	5	5	3	4	1	1	5	5	5	5	5	5	1	3	1	1	2	1	4	3	1	1	2	2

Websites for “ulcerative colitis, diet, children”:

1. https://www.crohnscolitisfoundation.org/youth-parent-resources/diet-and-nutrition

2. https://www.stlouischildrens.org/conditions-treatments/inflammatory-bowel-disease-ibd-center/education/diet-inflammatory-bowel-disease

3. https://www.massgeneral.org/children/inflammatory-bowel-disease/different-diets-for-ibd

4. https://pmc.ncbi.nlm.nih.gov/articles/PMC7922138/

5. https://www.childrensnational.org/get-care/health-library/ulcerative-colitis-in-children

6. https://www.crohnscolitisfoundation.org/patientsandcaregivers/diet-and-nutrition/what-should-i-eat

7. https://www.crohnscolitisfoundation.org/patientandcaregivers/diet-and-nutrition/special-ibd-diets

8. https://www.rch.org.au/kidsinfo/fact_sheets/Inflammatory_bowel_disease/

9. https://www.seattlechildrens.org/conditions/ulcerative-colitis/

10. https://www.crohnscolitisfoundation.org/sites/default/files/2020-03/diet-and-nutrition-brochure.pdf

11. https://www.cicra.org/what-is-ibd/diet-and-nutrition/

12. https://www.sciencedirect.com/science/article/pii/S2667009724000241

13. https://www.healthline.com/health/ulcerative-colitis/pediatric-ulcerative-colitis-diet

14. https://www.webmd.com/ibd-crohns-disease/ulcerative-colitis/ss/slideshow-uc-child-nutrition

15. https://www.seattlechildrens.org/healthcare-professionals/provider-news/treating-ibd-diet/

16. https://www.uhhospitals.org/services/digestive-health-services/conditions-and-treatments/small-and-large-intestine/inflammatory-bowel-disease/diet-guide

17. https://www.news-medical.net/news/20240205/High-quality-early-diet-linked-to-lower-IBD-risk-in-children.aspx

18. https://www.gastroenterologyadvisor.com/features/ibd-diet/

19. https://www.crohnscolitisfoundation.org/sites/default/files/legacy/assets/pdfs/diet-nutrition-2013.pdf

20. https://www.ucsfhealth.org/education/nutrition-tips-for-inflammatory-bowel-disease

21. https://www.choc.org/wp-content/uploads/2017/02/2-RypkemaL-RDsInPracticeGI.pdf

22. https://www.mycrohnsandcolitisteam.com/resources/pediatric-ulcerative-colitis-healthy-meal-ideas-for-kids

23. https://www.healthcentral.com/condition/ulcerative-colitis/pediatric-ulcerative-colitis-diet

24. https://www.niddk.nih.gov/health-information/digestive-diseases/ulcerative-colitis/eating-diet-nutrition

25. https://www.yalemedicine.org/conditions/pediatric-ulcerative-colitis

26. https://www.stanfordchildrens.org/en/topic/default?id=ulcerative-colitis-in-children-90-P02020

27. https://www.verywellhealth.com/ulcerative-colitis-7111961

28. https://crohnsandcolitis.org.uk/info-support/information-about-crohns-and-colitis/all-information-about-crohns-and-colitis/children-young-adults-and-students/supporting-your-child

29. https://www.mayoclinic.org/diseases-conditions/ulcerative-colitis/diagnosis-treatment/drc-20353331

30. https://www.mdpi.com/2072-6643/15/19/4194

31. https://www.healthline.com/health/ulcerative-colitis-in-children

32. https://www.healthline.com/health/ulcerative-colitis-take-control/treatment

33. https://www.webmd.com/ibd-crohns-disease/ulcerative-colitis/ulcerative-colitis-in-children

34. https://www.uptodate.com/contents/ulcerative-colitis-beyond-the-basics

35. https://www.childrenshospital.org/programs/inflammatory-bowel-disease-center/patient-resources/ulcerative-colitis-videos/nutrition

36. https://www.chop.edu/conditions-diseases/ulcerative-colitis-in-children

37. https://www.healthline.com/health/ulcerative-colitis-take-control/diet-plan-recipes

38. https://www.chop.edu/news/how-nutrition-can-support-children-ibd

39. https://www.mdpi.com/2072-6643/13/11/373
